# Using mass cytometry to probe the STAT signaling landscape in circulating immune cells in Rheumatoid Arthritis uncovers signaling dysregulation and correlation with disease activity

**DOI:** 10.3389/fmed.2025.1622537

**Published:** 2025-12-09

**Authors:** Claudia Macaubas, Batuhan Bayram, Noor Hussein, Astraea Jager, Kara L. Davis, Jonathan Graf, Mary Nakamura, Devy Zisman, Elizabeth D. Mellins

**Affiliations:** 1 Department of Pediatrics, Program in Immunology, Stanford University, Stanford, CA, United States; 2 Pediatric Hematology, Oncology, Stem Cell Transplant and Regenerative Medicine, Stanford University, Stanford, CA, United States; 3 Department of Medicine, University of California San Francisco, San Francisco, CA, United States; 4 Rheumatology Unit, Carmel Medical Center, Haifa, Israel; 5 The Ruth and Bruce Rappaport Faculty of Medicine, Technion-Israel Institute of Technology, Haifa, Israel

**Keywords:** Rheumatoid Arthritis, mass cytometry, STAT phosphorylation, whole blood, immune cells

## Abstract

**Introduction:**

The phosphorylation state of signaling proteins is a useful measure of cell function. The high dimensionality of mass cytometry (CyTOF) allows assessment of a large number of parameters using limited amount of material. By combining CyTOF high dimensionality with fixation of blood cells close to collection, cell phosphorylation patterns are preserved as close as possible to *in vivo* conditions. We used Cytof and whole fixed blood to investigate the phosphorylated forms of five Signal Transducers and Activators of Transcription - STATs - in Rheumatoid Arthritis (RA) patients and controls.

**Materials and Methods:**

We designed an antibody panel identifying 34 immune subpopulations combined with the phosphorylated (p) forms of Stat1, Stat3, Stat4, Stat5 and Stat6. Whole fixed blood from 21 RA patients and 10 healthy controls were analyzed by CyTOF. The frequency of immune cell subpopulations and the level of the pSTATs proteins were compared between the samples from RA patients and controls using Multiple Mann Whitney tests and clustering analysis. Correlation of the tested parameters with DAS28-ESR, DAS28-CRP and CDAI was examined.

**Results:**

Levels of pSTAT5 were elevated in several CD4 T cell subsets, and positively correlated with DAS28-ESR and DAS28-CRP. pSTAT1 levels were elevated in CD4 T cell subpopulations, as well as in some subsets of CD8 T cell subsets, NK cells, monocytes and Dendritic cells. Levels of pSTAT6 in several CD4 T cell subpopulations, including T regulatory cells, were lower in samples from RA patients compared to controls, and negative correlations between CDAI and pSTAT6 were found. The frequency of CD4 T cell effector memory cells expressing the chemokine receptors CCR2 and CCR5 was lower in samples from RA patients compared to controls.

**Conclusion:**

We found distinct pattern of STAT proteins phosphorylation associated with circulating immune cells in RA samples compared to healthy controls. These findings correlated with measures of disease activity. Analysis of phosphorylation patterns may be useful to understand disease pathology, and also as a marker of treatment response and disease outcome. By avoiding cell isolation, phosphorylation patterns in several cell subpopulation can be analyzed close to the *in vivo* cell profile.

## 1 Introduction

Phosphorylation of signaling proteins occurs upon stimulation by external stimuli, mostly cytokines. The activation of signaling pathways in individual cells is related to the capacity of the cells to respond to different stimuli. In this way, analysis of signaling cell status may be considered a proxy measure of cell function, as a “bridge between phenotypic and functional assays” as stated in Toney et al. (1), which usually require larger cell numbers.

Furthermore, to be able to assess the phosphorylated status of cells as close as possible to an *in vivo* situation would be very useful to understand disease biology and potentially to monitor patients’ treatment responses.

The high dimensionality of mass cytometry, also known as cytometry by time-of-flight (CyTOF), combines flow cytometry and mass spectrometry allowing assessment of overall heterogeneity and degree of similarity between subsets of immune cells population, based on a large number of parameters (2). Combining the high dimensionality of Cytof with the analysis of signaling using phospho flow cytometry (3) allows the characterization of the phosphorylation state of individual signaling proteins in a large number of cell subsets simultaneously, yielding a large amount of information about several cell subpopulations.

Additionally, by using blood fixed just after collection, the phosphorylation status of circulating cells close to *in vivo* status can be preserved. In this way, analysis of the phosphorylation status of large set of immune cells can be performed with significant less material than would be required for other functional and phosphorylation assays, and without the need for additional steps for cell isolation.

In this study, we used CyTOF to analyze the phosphorylation status of five signal transducer and activator of transcription - STATs - proteins in 34 cell populations in Rheumatoid Arthritis (RA). To do this analysis, we used whole blood what was fixed shortly after drawing, preserving the signaling landscape as close as possible to the “*in vivo*” condition. In this way, our study has a significant advantage over studies using cells obtained after multistep isolation, which may alter the signaling status of the cells (4), or studies that analyzed only a few parameters using flow cytometer (5). RA is a common autoimmune disease of unknown etiology affecting joints and other organs, and with significant involvement of circulating immune cells (6, 7). There is growing interest in targeting transcription factors for RA therapy (8). Many mediators involved in RA are regulated by transcription factors including the JAK (Janus kinase)/STATs pathway. Among the seven STAT members, STAT1 -3, -4, -5 and -6 have been found to be abundantly expressed in RA (8). Members of the JAK/STAT family are activated by several cytokines that have been shown to be produced in a dysregulated manner in RA (9), affecting multiple cell types that play important role in RA pathology, including lymphocytes, macrophages and fibroblast-like synoviocytes. Supplementary Table 1 provides further information regarding STATs activating cytokines and the relevance of these STATs proteins to RA. Targeting of JAK, that are recruited to membrane receptors and subsequently phosphorylated the STATs proteins, showed the potential of this approach to treat RA (10). Furthermore, direct target of STATs protein, such as STAT3 may be even more efficacious with potential less adverse effects on immune responses (11, 12). Response to treatment has been shown to be associated with levels of phosphorylation response of STAT1 and STAT6 in circulating leukocytes from RA patients (13). However, several of these studies analyzed cells after several steps of isolation, or analyzed a limited number of cell populations. In this study, we analyzed cells from blood fixed shortly after collection to demonstrate the potential usefulness of this approach to capture the signaling landscape in circulating immune cells.

## 2 Materials and methods

### 2.1 Participants information

Patients with Rheumatoid Arthritis (RA) who meet the American College of Rheumatology (ACR) classification criteria (14) were recruited from University of California, San Francisco (UCSF) rheumatology clinic. The research was performed with approval from the Institutional Review Board (IRB) from UCSF (IRB number 13-12711) and Stanford University (IRB number 33260). A total of 21 RA patients were included in the study; fifteen patients (71%) were classified as in remission or mild disease (DAS28 CRP < 3.2) and six (29%) as moderate or high activity disease (DAS28 CRP > 3.2). Summary of demographic and clinical information for the RA patients is in Table 1, with detailed patient information in Supplementary Table 2. With the exception of one patient in remission, all others were on medication (Table 1). Ten healthy adult volunteers were recruited through the Stanford Blood Center (SBC) (Table 1).

**TABLE 1:** Rheumatoid Arthritis patients and controls information.

	Rheumatoid Arthritis patients	Control subjects
Number	21	10
Age (years)^1^	61.4 ± 14.18	61.8 + 8.32
Sex female, %	18 (86%)	10 (100%)
Disease duration (years)	11.8 ± 10.36	NA
ESR (mm/hr)	25.00 ± 12.54	Normal range: Women > 50 years: <30 Men > 50 years: <20 (52)
CRP (mg/dL)	4.76 ± 4.83	Normal value: <0.3 Normal or minor elevation: 0.3–1.0 (53)
Rheumatoid factor (range)	Negative −1280	In up to 4% of healthy individuals (54)
Anti-CCP titer	169.5 ± 75.71	Healthy range: negative
DAS 28-ESR	3.36 ± 0.89	NA
DAS 28-CRP	2.66 ± 0.83	NA
CDAI	10 ± 8.24	NA
Tender joints	0.86 ± 1.46	NA
Swollen joints	3.33 ± 6.03	NA
**Medications**		
*cDMARDs* ^2^		
MTX	16	
Sulfasalazine	1	
*bDMARDs* ^3^		
Abatacept	2	
Certolizumab	2	
Etanercept	4	
Adalimumab	4	
None	9	
Prednisone	12	

^1^Continuous variables are presented as mean ± SD. ^2^c-DMARDS, conventional disease-modifying antirheumatic drugs; ^3^b-DMARDS, biological disease-modifying antirheumatic drugs. For medication, the number of patients refers to the total number of patients on the particular medication in each group; NA, not applicable.

### 2.2 Samples

For RA patients, heparinized whole blood collected in vacutainer tubes was dispensed into Smart Tubes (Smart Tube Inc.), which contain Smart Tube fixative (proprietary formula) for 1 ml of blood. Heparinized blood samples from healthy donors from the SBC were mixed at the sample proportion with the same Smart Tube fixative. After addition of fixative, samples were incubated at room temperature (RT) for 10 min and transferred to −80°C storage. Samples were kept at −80°C until thawing. The fixed whole blood was thawed in a 4°C cold room as described in Macaubas et al. (15). Briefly, following complete thawing, samples were mixed 1:5 with 1 × Smart Tube Thaw-Lyse Buffer (Smart Tube) and centrifuged at 600 × *g* for 8 min at RT. The resulting pellet was sequentially washed with Thaw-Lyse Buffer in a total of 3 times, followed by 2 washes with 25 ml of thaw Lyse Buffer 2 (Smart Tube). The final pellet re-suspended in 1.6 ml of 0.22 μm filtered CyFacs buffer [1 x PBS - CyPBS (Rockland), 1% bovine serum albumin (Millipore Sigma) and 0.05% sodium azide (Millipore Sigma) in Milli-Q water (Invitrogen)] and stored at 4°C overnight in a 96 well deep well polypropylene plate (VWR).

### 2.3 Antibodies

The antibody panel is shown on Supplementary Table 3. Antibodies clones were selected based on showing appropriate staining for fixed cells. We used fixed blood from two additional healthy adult controls to titrate the antibody panel. These two control samples were not included in the group of 10 healthy volunteers that were compared to RA patients. Antibodies were freshly combined for each experiment in a volume of 50 μl per sample. The total amount was filtered using a 0.1 μm Durapore PVDF filter (Millipore Sigma) at 14,000 × g for 5 min at RT.

### 2.4 Staining and mass cytometry

The staining was performed as described in Macaubas et al. (15), with modifications. Briefly, Fc receptors were blocked using 5 μl of Human TruStain FcX™ (BioLegend) for 10 min, followed by addition of the antibody cocktail for surface antigens (Supplementary Table 3). The deep well plate was incubated for 30 min at RT, followed by washing with CyFacs Buffer and fixation with 1.6% of formaldehyde (Thermo Scientific) for 10 min at RT, followed by 2 washes with CyPBS. Cells were then permeabilized with 90% methanol (Millipore Sigma), on ice for 30 min, followed by 2 washes with CyPBS, followed by addition of antibodies to intracellular antigens (Supplementary Table 3), and incubation for 30 min at RT. Cell DNA content was identified by incubation with 0.125 nM iridium (191Ir and 193Ir) (Cell-ID™ Intercalator-Ir, Fluidigm/Standard BioTools), for 20 min at RT, in a volume of 300 μl. Cells were then washed 2 times with CyFacs buffer and kept at 4 °C overnight. Next day, cells were washed 2 times with 0.22 μm filtered Milli-Q water; cells were counted before the last wash. A Helios mass cytometer (Fluidigm/Standard BioTools) was used for sample acquisition, performed as described in Macaubas et al. (15).

### 2.5 Mass cytometry data analysis

Cell subpopulations were manually determined using FlowJo version 10.10.0 (FlowJo, LLC). Supplementary Figure 1 shows the gating strategy. Briefly, cell events (intact cells) were identified as Ir191/193 double positive events, and doublets were excluded on the basis of higher DNA content (Ir191) and longer event length. Immune cell frequencies are expressed as frequency of mononuclear cells, defined as CD45 + , CD66b- cells, except for granulocytes and granulocytic myeloid-derived suppressor cells (G-MDSC) that are expressed as frequency of total leukocytes. The gating strategy from Ando et al. was mostly used (16), and modifications are noted on Supplementary Figure 1. For levels of phosphorylated signaling proteins, the signal intensity from a given cell subpopulation was measured; the geometric mean (gmean, as defined by FlowJo software) of the signal intensity for each value was expressed as the hyperbolic arcsine of the gmean divided by a cofactor parameter (value = 5) (arcsinh transformed).

### 2.6 Statistical analysis

Differences between parameters from RA and control groups were analyzed using unpaired Multiple Mann Whitney tests, and resulting *p*-values were corrected using the Benjamini-Hochberg procedure to control the False Discovery Rate [FDR(q)]. Correlations were performed using Spearman correlation. All analyses were done using GraphPad Prism version 10.

### 2.7 Heatmaps

Heat maps were created using the open-access visualization and analysis software Morpheus, available at https://software.broadinstitute.org/morpheus/. For Hierarchical Clustering, the distance metric used for clustering was based on one minus the Spearman rank correlation coefficient. The maximum and the minimum values in each row were used to convert values to color.

## 3 Results

### 3.1 Blood immune cell subpopulations distribution are mostly similar between RA patients and controls

**FIGURE 1:**
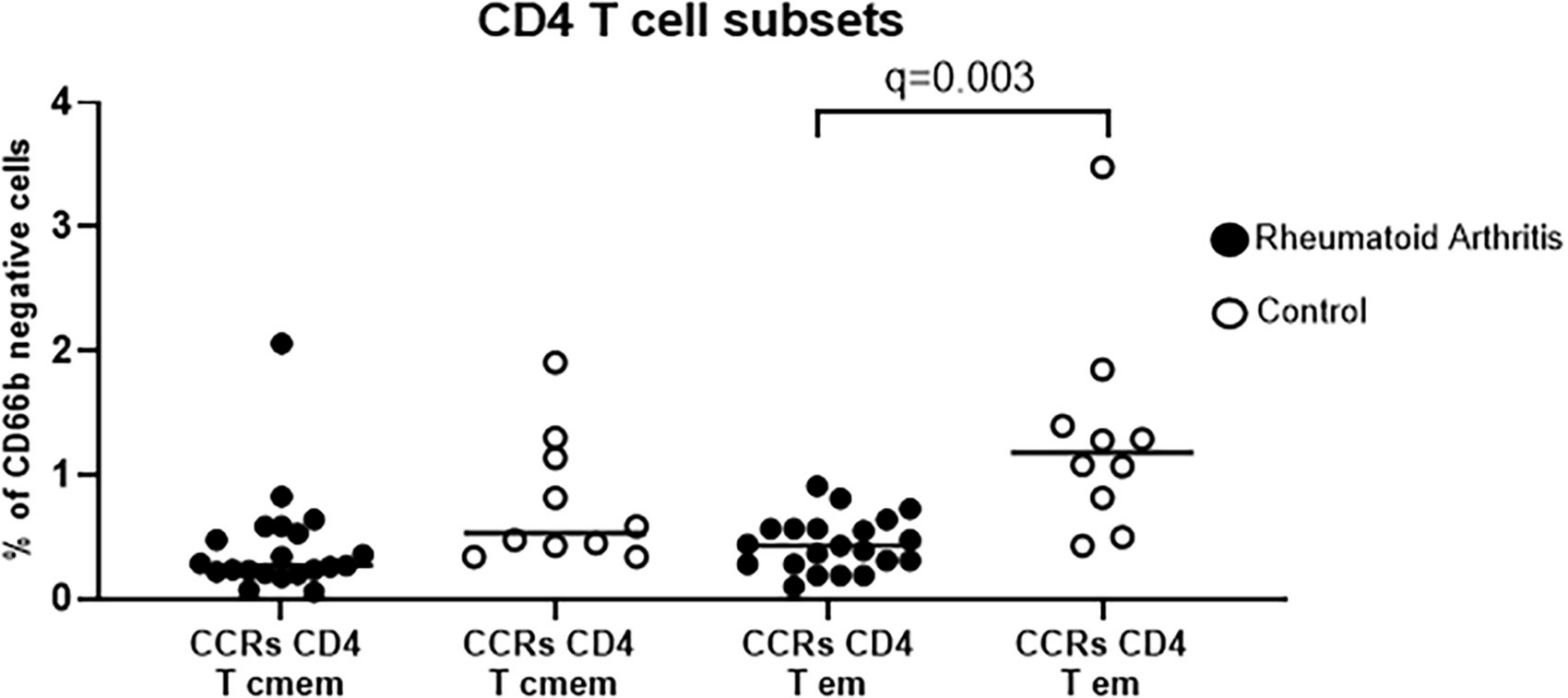
Frequency of circulating CCR5 + CCR2 + (CCRs) CD4 T cell effector memory (em) subset is lower in blood from Rheumatoid Arthritis (*n* = 21) patients compared to controls (*n* = 10), while frequency of circulating CCR5 + CCR2 + (CCRs) CD4 T cell central memory (cmem) subset is similar between RA and controls. Middle line represents the median. Comparisons between all 34 cell subpopulations from RA and control groups were performed using multiple Mann-Whitney tests, and the resulting *p*-values were corrected using the Benjamini-Hochberg procedure to control the False Discovery Rate [FDR(q)]. Comparisons between CCRs CD4 cmem from RA patients and controls were deemed non-significant.

We assessed the frequency of 34 immune subpopulations in from the blood of RA and controls subjects (Supplementary Figures 2–5). After correction for multiple testing [FDR(Q) = 1%], the only significant difference was the frequency of CD4 T cell effector memory (CD4 + T cell CD45RO + RA- CD62L-), expressing the chemokine receptors CCR2 and CCR5 (Supplementary Table 4). This population was lower in the blood of RA patients compared to controls, while frequency of CD4 T cell central memory cells expressing the chemokine receptors CCR2 and CCR5 was similar between the two groups (Figure 1).

### 3.2 Phosphorylation patterns are distinct between circulating immune cells from RA patients and controls

**FIGURE 2:**
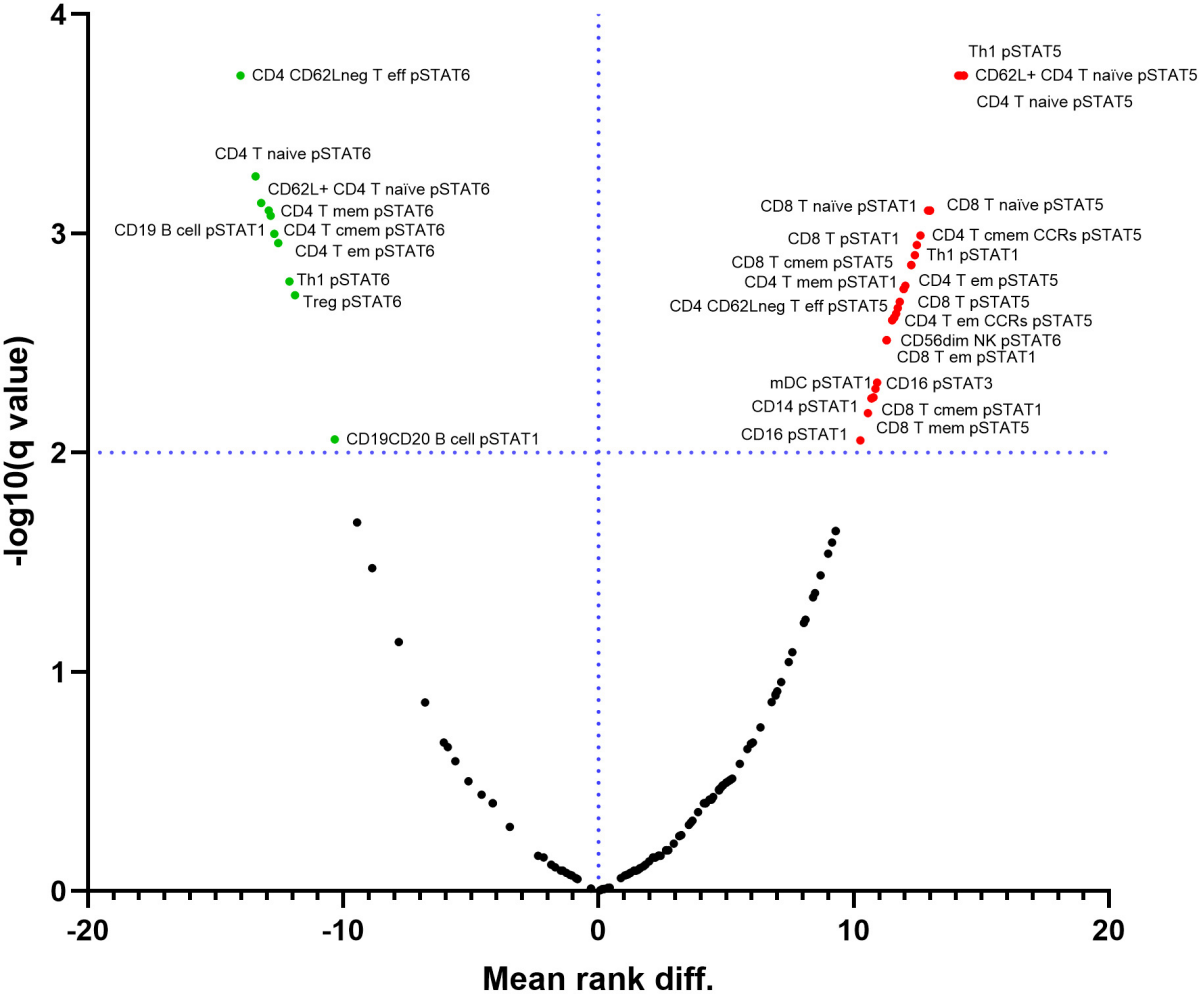
Differential expression of phosphorylated STATs proteins in immune cell subpopulations between samples from Rheumatoid Arthritis (RA) and controls. Red points on the upper right quadrant represent phosphorylated STATs with higher expression in cells from RA patients compared to controls. Green points on the upper left quadrant represent phosphorylated STATs with lower expression in cells from RA patients compared to controls. Black points below the dotted line represent values without statistically significant changes. Dotted line represents the –log10 of the False Discovery Rate (FDR) level (q) = 1%.

We analyzed the baseline levels of five phosphorylated STATs proteins (pSTAT1, pSTAT3, pSTAT4, pSTAT5 and pSTAT6) in all 34 immune subpopulations. At FDR = 1%, several differences were observed in the phosphorylation pattern from circulating blood immune cells between RA patients and controls (Figure 2 and Supplementary Table 5).

**FIGURE 3:**
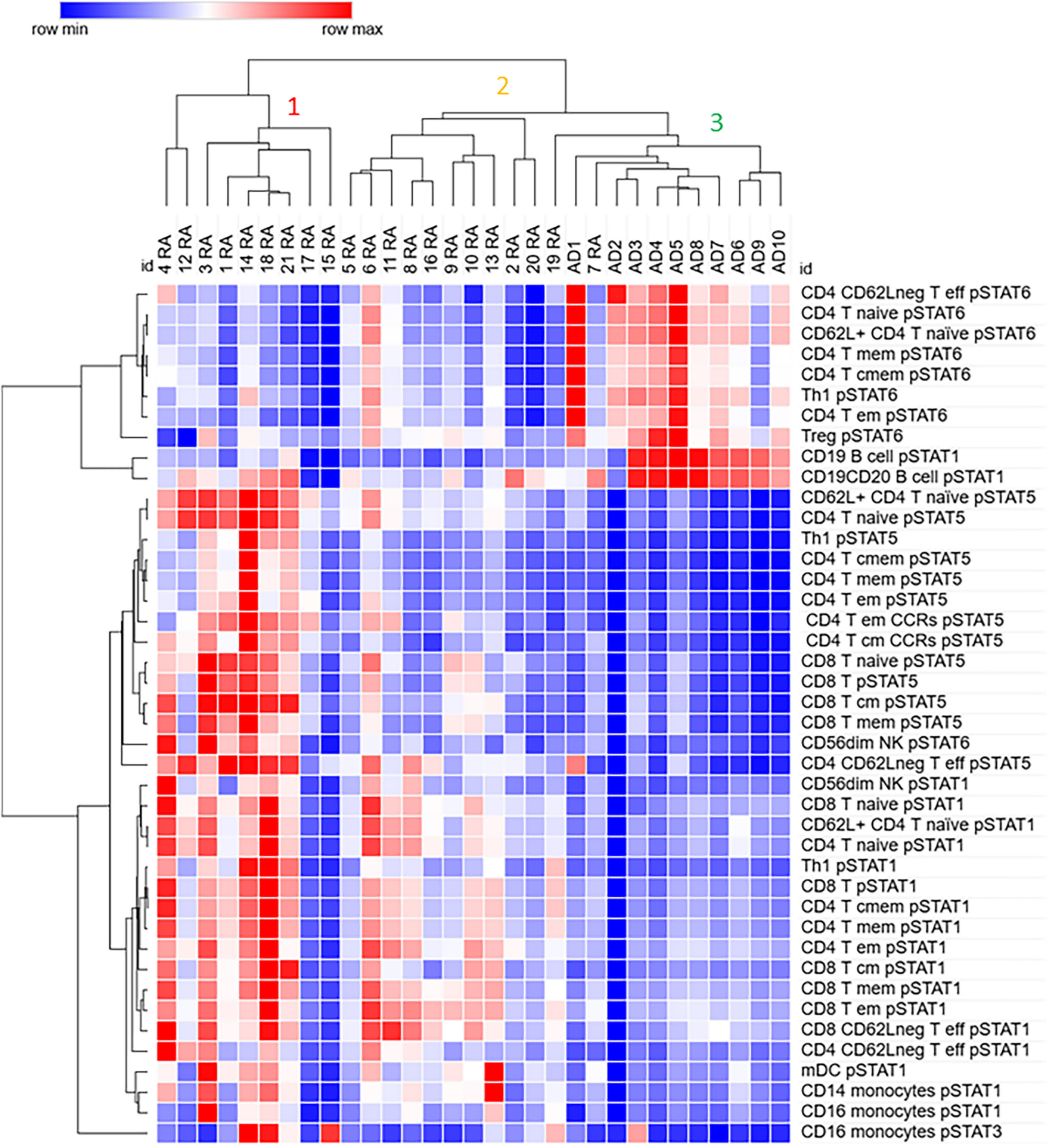
Differentially expressed phosphorylated STATs proteins in several immune subpopulations cluster samples from Rheumatoid Arthritis (RA) away from control (Adult donor, AD) samples. Values for pSTATs proteins for listed cell subpopulations were used for hierarchical clustering using the analysis software Morpheus as described in Materials and Methods.

We used Hierarchical clustering to analyze the relationship between the differentially expressed phosphorylated STATs proteins in RA and control samples. The samples clustered in 3 clusters; the cluster 1 contains 5 (out of 6) moderate RA samples and 4 mild ones. This cluster is characterized by low pSTAT6 in several CD4 T cells subsets compared to cluster 3, where all control samples are, and higher pSTAT5 levels in several CD4 and CD8 T cell subsets compared to the clusters 1 and 2 (Figure 3).

Cluster 2 contains only mild RA samples, and it is closer to cluster 3, which contains all control samples, than to the RA cluster 1. However, as cluster 1, cluster 2 shows lower pSTAT6 in several CD4 T cells subsets compared to samples in cluster 3. In contrast to cluster 1, cluster 2 shows lower levels of pSTAT5 in the cell populations analyzed. Both cluster 1 and 2 show higher levels of pSTAT1 in several CD4 and CD8 subsets compared to cluster 3. Some patients in cluster 1 also show elevated levels of pSTAT1 in mDC and monocytes, as well as pSTAT3 in monocytes (CD16 +).

Cluster 3, in addition to all control samples, contains one RA remission sample (patient 19) as well as a mild RA sample. In addition to high levels of pSTAT6 compared to clusters 1 and 2, cluster 3 also shows high level of pSTAT1 in B cells.

These results suggest that elevated levels of pSTAT5 and pSTAT1, and lower levels of pSTAT6 in subsets of CD4 T cells, as well as in CD8 T cells, may be important in distinguishing RA from control samples.

**FIGURE 4:**
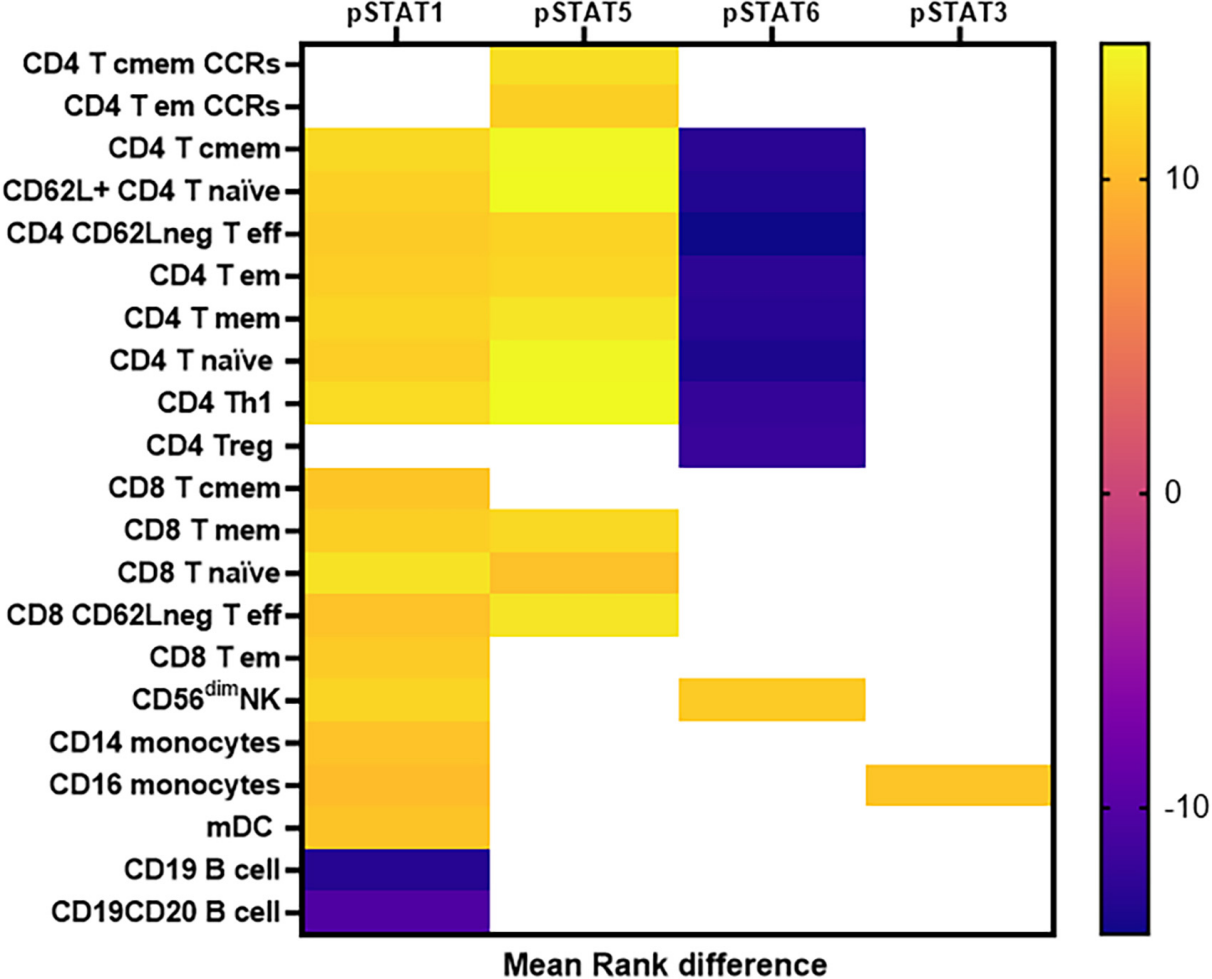
Heat map shows the expression pattern of differentially expressed phosphorylated STATs proteins in distinct cell subsets. Values are expressed as mean rank difference between RA patients and controls values. Eff, effector; em, effector memory; cmem, central memory; mem, memory; mDC, myeloid Dendritic cell.

To better visualize the relationship between the level of pSTATs and the cell subsets, we used the mean rank difference between the pSTATs levels RA and control group (Supplementary Table 5) to build a heat map. As can be seen in Figure 4, only some CD4 T cell subsets show significant alterations in multiple pSTATs, with higher levels of pSTAT1 and pSTAT5 in RA, and lower levels of pSTAT6 in RA patients compared to controls. However, CD4 T cell memory subsets expressing the chemokine receptors CCR2 and CCR5 show only higher pSTAT5 levels in cells from RA patients.

CD8 T cell subsets, on the other hand, show only higher levels of pSTAT1, with only a few subsets expressing higher pSTAT5 in RA compared to controls. The major NK cell population, NK CD56*^dim^* cells, are the only subset expressing higher pSTAT6 in RA compared to controls (in addition to higher pSTAT1).

Monocytes and dendritic cells show higher levels of pSTAT1, and pSTAT3 in CD16 monocytes only, in RA patients compared to controls.

### 3.3 Levels of pSTAT5 and pSTAT6 in several lymphocytes’ subpopulations correlate with disease activity

**FIGURE 5:**
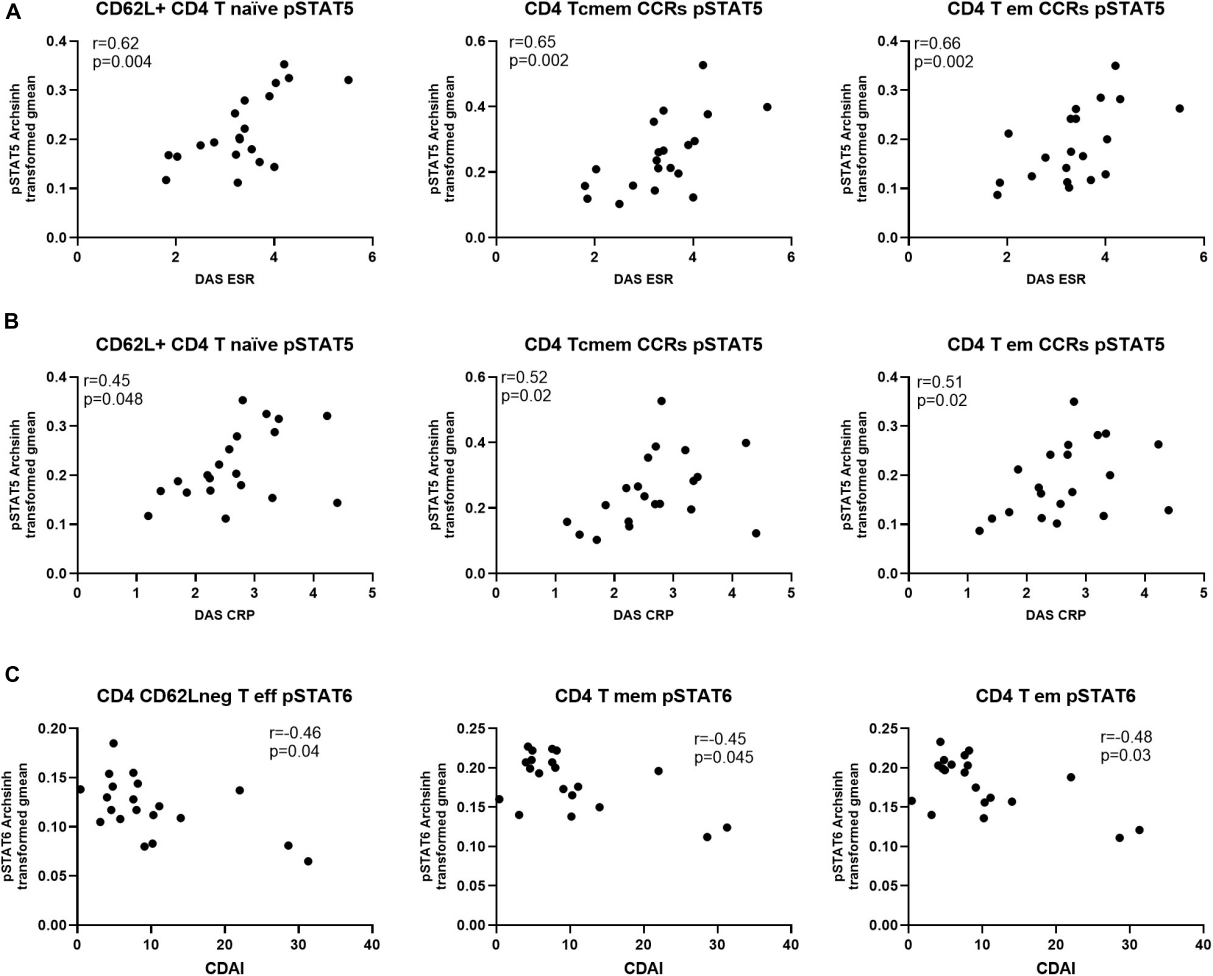
Levels of pSTAT5 in CD4 T cell subsets are positively correlated with indicators of disease activity, while pSTAT6 is negatively correlated. Spearman correlation analysis between level of pSTAT5 and (A) DAS-ESR, (B) DAS-CRP and (C) CDAI, *n* = 20. CCRs, CCR5 + and CCR2 + ; cmem, central memory; em, effector memory; eff, effector.

Next, we investigated if the levels of differentially expressed phosphorylated STATs proteins correlated with measures of disease activity, specifically Disease Activity Score-28 - DAS28-ESR, DAS28-CRP and Clinical Disease Activity Index (CDAI). The analysis showed that only levels of pSTAT5 correlated positively and significantly with DAS28-ESR and -CRP. Furthermore, with the exception of CD8 T central memory cells, all pSTAT5 correlations involved CD4 T cell subsets (Table 2). Another observation was that, while statistically significant DAS28-ESR positive correlations with levels of pSTAT5 were found with several CD4 T cell subsets, DAS28-CRP showed lower levels of correlation, with significant correlations with levels of pSTAT5 in only three CD4 subsets (Figure 5 and Table 2). On the other hand, correlations with CDAI, which does not include a marker of inflammation, showed significant negative correlation between CDAI and levels of pSTAT6 in three CD4 T cell subsets (Figure 5 and Table 2).

**TABLE 2:** Spearman correlation values for DAS28-ESR, DAS28-CRP, and CDAI and level of phosphorylated STATs in differentially expressed cell subpopulations.

Disease activity index	Cell subpopulations												
	CD4 T cmem CCRs	CD4 T em CCRs	CD4 T cmem	CD62L+ CD4 T naïve	CD4 CD62Ln T eff		CD4 T em		CD4 T mem		CD4 T naïve	Th1	CD8 T cmem
* pSTAT*	*5*	*5*	*5*	*5*	*6*	*5*	*5*	*6*	*5*	*6*	*5*	*5*	*5*
DAS28	0.66	0.66	0.60	0.62	−0.13	0.46	0.54	−0.22	0.54	−0.10	0.59	0.46	0.45
ESR	0.00	0.00	0.01	0.00	0.58	0.04	0.01	0.35	0.02	0.69	0.01	0.04	0.05
DAS28	0.52	0.51	0.41	0.45	−0.20	0.39	0.41	−0.32	0.38	−0.16	0.44	0.27	0.28
CRP	0.02	0.02	0.07	0.05	0.41	0.09	0.07	0.17	0.10	0.51	0.05	0.25	0.23
CDAI	0.34	0.43	0.33	0.30	−0.46	0.27	0.33	−0.48	0.28	−0.45	0.25	0.21	0.26
	0.14	0.06	0.16	0.20	0.04	0.24	0.16	0.03	0.23	0.05	0.29	0.37	0.26

Numbers represent r (top) and P (bottom) values; significant correlations are in bold. cmem, central memory; CCRs, CCR5 and CCR2 chemokine receptors; em, effector memory; CD62Ln, CD62L negative; eff, effector.

## 4 Discussion

In this study, we examined 34 immune subpopulations in blood cells from Rheumatoid Arthritis (RA) patients. The blood was fixed shortly after collection, allowing examination of cell profiles close to the *in vivo* condition. First, we compared if the frequencies of these immune subpopulations were distinct between RA patients and controls. We found differences only regarding the CCR2 + CCR5 + CD4 T cell effector memory subset, with lower levels of these cells in RA patients. Medication usage may affect the expression of chemokine receptors. The effects, however, are complex. For example, it has been found that methotrexate (MTX), which almost all patients were taking at time of blood sampling, has been shown to affect CCR2 expression in monocytes but to be more associated with disease activity in CD4 + T cells (17). Anti-TNF medication, which was also largely used, has been shown to be associated with lower expression of CCR2 in CD3 + T cells, while CCR5 was affected to a lower degree (18). While we cannot exclude that medications may have contributed, at least in part, to the decrease of the CCR2 + CCR5 + cells effector memory T cells in RA patients, we did not observe a significant decrease in other populations that also coexpress CCR2 and CCR5. T effector memory cells produce pro-inflammatory cytokines such as IFNƴ and TNF and the expression of the chemokines receptors CCR2 and CCR5 allow these cells to rapidly migrate to inflammatory sites, attracted by CCL2 (MCP1) and CCL5 (RANTES), respectively (19). CCL2, one of the ligands of CCR2, has been shown to be produced by the chondrocytes and synovial fibroblasts in RA (20). The T cells expressing CCR2 and CCR5 may have migrated from the circulation into the inflamed tissues in RA patients. In fact, T cell clones isolated from RA patients coexpressing CCR2 and CCR5 have shown high migratory capacity in *in vitro* assay of cell migration (21), and the majority of T cells in the joint express CCR2 and CCR5 (22). Based on previous studies [reviewed in Künzli and Masopust (23)], we speculate that these CCR2 + CCR5 + effector memory cells in RA patients may have reactivated, left the circulation and migrated to inflamed tissues, while central memory cells continue to recirculate, and whose level are indeed similar between RA and controls. CCR5 + cells have been shown to accumulate in the synovial compartment, and that these cells produce MMP-9 and osteopontin, two mediators that are implicated in RA (20). Much remains to be understood about these cells. Recent work identified T resident memory cells (TRM), which can be CD4 or CD8, in synovium in remission, and some evidence suggest that these cells, or their descendants, may leave the tissues and contribute to the population of T cell central and effector memory (24, 25).

The finding that only the level of CCR2 + CCR5 + CD4 T cell effector memory subset was different between RA patients and controls may be related to the fact that the RA patients were mostly well controlled clinically.

In contrast to the mostly similar cell distribution between RA patients and controls, several differences were found in the phosphorylation levels of pSTATs in several cell subpopulations. These findings may suggest that, at least in a well-controlled patient group, examination of cell distribution alone may not be sufficiently informative to distinguish between controls and patients’ groups. Analysis of parameters like phosphorylated proteins provide another layer of information about functional status, and as we show, can be executed with small blood volumes with a great degree of depth and keeping cell characteristics as close as possible to *in vivo* conditions.

We examined levels of phosphorylated STAT1, -3, -4, -5 and -6 in 34 cell populations from blood. Several pSTATs were differentially expressed between RA patients and controls, and hierarchical cluster analysis showed that the pSTATs expression pattern clustered RA samples away from controls. This complex pattern of phosphorylation among diverse cell populations is likely associated with several factors, such as diverse response of cells to systemic and local stimuli, level of cytokine receptors, negative feedback mechanisms such as SOCS proteins, and level of unphosphorylated STATs, among others (26–32). The cluster analysis showed that lower levels of pSTAT6 were observed in subsets of CD4 T cells from RA patients compared to controls. STAT6, activated by IL-4 and IL-13, is a key transcription factor involved in the initiation and expansion of Th2 response, through its role as a mediator of IL-4 response. In RA, Th2 conditions are considered to be protective, hence lower levels of pSTAT6 in RA compared to controls may suggest that anti-inflammatory responses are reduced, and Th2-type response may be part of an initial regulatory role in RA, and lost once disease is established (33). In experimental arthritis, Th2-type response has been shown to have anti-inflammatory and anti-osteoclastogenic properties (33). However, the role of pSTAT6 in RA may be more complex, with decreasing pSTAT6 from baseline in lymphocytes associated with good response to treatment (13). Subsets of Th2 cells (for example, CXCR3 + Th2) may also play a pathogenic role in synovial inflammation in RA (34).

Levels of pSTAT6 were also lower in T regulatory (Treg) CD4 T cells in RA compared to controls. STAT6 is downstream of IL-4 and IL-13, and it has been shown that in the absence of IL-4, Treg suppression activity is decreased (35). This suggests that Treg cells in RA patients might be defective (36), as in conditions of STAT6 deficiency, induction and functionality of Tregs may be impaired (37).

Levels of pSTAT6 were higher in CD56^dim^ NK cells in RA compared to controls. Circulating NK cells in RA patients have been found to have reduced cytotoxicity and capacity to produce IFNƴ (38). NK cells from Stat6 deficient mice have been shown to have higher cytotoxic activity. The effects of IL-4 on NK cells is still unclear, with reports of reduced as well as increased cytotoxicity activity (39). Nevertheless, there are indications that increased STAT6 activity is associated with reduced NK function.

Higher levels of pSTAT1 and pSTAT5 in cells from patients RA compared to controls were found in the same CD4 subpopulations that showed lower levels of pSTAT6 in RA, with the exception of Treg cells. STAT1 and STAT5 are associated with Th1-like conditions, which are involved in synovial inflammation and bone metabolism and immune responses (33). Several CD8 subpopulations expressed higher levels of pSTAT1 and a few pSTAT5, but not lower pSTAT6, in RA compared to controls.

STAT1, which is activated by pro-inflammatory cytokines IFNƴ and TNF, and also IL-2 among several others (8), promotes the differentiation of Th1 pro-inflammatory cells. The cytokine produced by these Th1 cells are involved in synovial inflammation, cartilage destruction and bone erosion (33). Of course, Th17 cells are also involved in proinflammatory responses in RA (33), although we did not find evidence in our study related to Th17 cells. STAT5, activated by cytokines of the IL-2 family among others, and although associated with Th1 pathway, also participates in the development of the Th2 and Th17 pathways.

We also found that circulating myeloid DCs from RA patients show higher levels of pSTAT1 compared to controls. STAT1 is associated with Th1 cell priming as well as DC maturation (40). Similarly, CD14 + (classical) and CD16 + (non-classical) monocytes showed higher levels of pSTAT1 in RA compared to controls. These results agree with a previous study that described elevated pSTAT1 in monocytes from RA patients, isolated from PBMC (41), and where monocytes were defined by size and granularity only. In our study, we confirm this result in specific monocyte subpopulations without isolation.

We also observed that CD16 + monocytes from RA patients showed higher levels of pSTAT3 than controls. Elevated levels of pSTAT3 in circulating T cells and monocytes from RA patients with active disease have been described (42). CD16 + monocytes are described as “patrolling monocytes” and may be able to migrate to tissues (43), and activation of the STAT3 pathway may contribute to cell migration (44).

Although several alterations in pSTATs levels were found between samples from RA patients and controls, when we examine correlations with disease activity, only levels of pSTAT5, mostly in some CD4 subsets, were found to be positively correlated with indicators of disease activity DAS28-ESR and DAS28-CRP. DAS28-CRP was significantly positively correlated with three subsets that also correlated with DAS28-ESR, CD4 + CD62L + naïve cells, and CD4 central memory and effector memory, both carrying CCR2 and CCR5 receptors. The CD4 effector memory CCR2 + CCR5 + also shows differential levels between samples from RA patients and controls, suggesting that this subset may play an important role in RA pathology. Although higher levels of pSTAT1 was observed to be differentially expressed in several cell subsets between RA patients and controls, we did not observe any significant correlation with disease activity. We also examined correlations with Clinical Disease Activity Index (CDAI) and we found significant negative correlations with pSTAT6 in CD4 T cell memory and effector memory and CD62L negative effector cells. The CDAI score is a composite index that includes symptoms and physical examination, and does not include a marker of inflammation. CDAI integrates various aspects of the disease and may provide a broader assessment of disease activity. Our findings suggest that higher levels of pSTAT6 in CD4 T cells may be related to factors promoting improved clinical status, while higher pSTAT5 levels are associated with higher levels of inflammation as expressed by ESR and CRP. These effects may be mediated by the cytokine microenvironment, which CD4 T cell subsets especially responsive to it, as found by divergent expression patterns affecting pSTAT5 and pSTAT6. pSTAT1, although differentially expressed in several cell subsets, did not correlated with measures of disease activity, and may be more associated with immunoregulatory roles (33).

Our study has several limitations. We examined a small number of RA patients, with a significant part comprising of well-controlled patients. As such, differences between the RA patients and controls may have been attenuated. However, several studies suggest that a large proportion of RA patients can exhibit active inflammation in the presence of normal levels of inflammatory markers. Specifically, studies show that evidence of clinical disease can be found in patients with normal ESR or CRP (45, 46), and also that in over 50% of patients classified as in remission by DASR28-ESR or -CRP, evidence of persistent synovitis can be found by ultrasound and magnetic resonance imaging studies (47–50). A recent study also showed that in at risk individuals, immunological signs of systemic inflammation have been found before evidence of active synovitis (51). Nevertheless, the lack of patients with higher disease activity is another limitation of our study. Additionally, while it is likely that treatment affects the phosphorylation pattern of immune cells, the small number and patient heterogeneity did not allow an in-depth analysis of these variables. Future studies including a large number of patients and controls, allowing for stratification by specific medications and/or disease activity will be very informative. Another alternative include longitudinal studies following patients’ response to medications, especially medications targeting signaling pathways. These studies, using a similar approach described here, will likely further uncover disease immunopathology and help improve to understand response to treatment.

In summary, examination of phosphorylated patterns of several STATs proteins in whole fixed blood from RA patients shows a Th1-like pattern of phosphorylation in several subsets of T cells (both CD4 and CD8) and decreased Th2-like response in comparison to controls. Monocytes and DCs also show a pattern of activation as seen by level of phosphorylated pSTATs. Levels of pSTAT5 in some CD4 subsets are positively correlated with disease activity, while levels of pSTAT6 are negatively correlated with CDAI. Elevated pSTAT3 was observed in CD16 + (non-classical) monocytes. These findings suggest that the approach used in this study may be helpful to examine cellular function in RA and other diseases as close as possible to an *in vivo* state.

## Data Availability

The raw data supporting the conclusions of this article will be made available by the authors, without undue reservation.

## References

[1] Toney NJ Schlom J Donahue RN. Phosphoflow cytometry to assess cytokine signaling pathways in peripheral immune cells: potential for inferring immune cell function and treatment response in patients with solid tumors. *J Exp Clin Cancer Res*. (2023) 42:247. 10.1186/s13046-023-02802-1 37741983 PMC10517546

[2] Leipold MD Newell EW Maecker HT. Multiparameter phenotyping of human PBMCs using mass cytometry. *Methods Mol Biol*. (2015) 1343:81–95. 10.1007/978-1-4939-2963-4_7 26420710 PMC4748856

[3] Krutzik PO Nolan GP. Intracellular phospho-protein staining techniques for flow cytometry: monitoring single cell signaling events. *Cytometry A*. (2003) 55:61–70. 10.1002/cyto.a.10072 14505311

[4] Ptacek J Hawtin RE Sun D Louie B Evensen E Mittleman BB Diminished cytokine-induced Jak/STAT signaling is associated with rheumatoid arthritis and disease activity. *PLoS One*. (2021) 16:e0244187. 10.1371/journal.pone.0244187 33444321 PMC7808603

[5] Dreo B Muralikrishnan AS Husic R Lackner A Brügmann T Haudum P JAK/STAT signaling in rheumatoid arthritis leukocytes is uncoupled from serum cytokines in a subset of patients. *Clin Immunol*. (2024) 264:110238. 10.1016/j.clim.2024.110238 38729230

[6] Jang S Kwon EJ Lee JJ. Rheumatoid Arthritis: pathogenic Roles of Diverse Immune Cells. *Int J Mol Sci*. (2022) 23:905. 10.3390/ijms23020905 35055087 PMC8780115

[7] Rana AK Li Y Dang Q Yang F. Monocytes in rheumatoid arthritis: circulating precursors of macrophages and osteoclasts and, their heterogeneity and plasticity role in RA pathogenesis. *Int Immunopharmacol*. (2018) 65:348–59. 10.1016/j.intimp.2018.10.016 30366278

[8] Balendran T Lim K Hamilton JA Achuthan AA. Targeting transcription factors for therapeutic benefit in rheumatoid arthritis. *Front Immunol*. (2023) 14:1196931. 10.3389/fimmu.2023.1196931 37457726 PMC10339812

[9] McInnes IB Buckley CD Isaacs JD. Cytokines in rheumatoid arthritis - shaping the immunological landscape. *Nat Rev Rheumatol*. (2016) 12:63–8. 10.1038/nrrheum.2015.171 26656659

[10] Kiełbowski K Plewa P Bratborska AW Bakinowska E Pawlik A. JAK inhibitors in rheumatoid arthritis: immunomodulatory properties and clinical efficacy. *Int J Mol Sci*. (2024) 25:8327. 10.3390/ijms25158327 39125897 PMC11311960

[11] Oike T Sato Y Kobayashi T Miyamoto K Nakamura S Kaneko Y Stat3 as a potential therapeutic target for rheumatoid arthritis. *Sci Rep*. (2017) 7:10965. 10.1038/s41598-017-11233-w 28887478 PMC5591217

[12] Gardino A Bifulco N Hunt J Vaswani R Park D Metz P REX-7117 is a highly potent and selective oral STAT3 inhibitor that demonstrated potential efficacy and safety differentiation versus JAK/TYK2 targeting in preclinical models of inflammatory arthritis. *Arthritis Rheumatol.* (2024) 76(Suppl 9):1964–5. Available online at: https://acrjournals.onlinelibrary.wiley.com/doi/epdf/10.1002/art.42992

[13] Kuuliala K Kuuliala A Koivuniemi R Kautiainen H Repo H Leirisalo-Repo M. STAT6 and STAT1 pathway activation in circulating lymphocytes and monocytes as predictor of treatment response in rheumatoid arthritis. *PLoS One*. (2016) 11:e0167975. 10.1371/journal.pone.0167975 27942004 PMC5152841

[14] Aletaha D Neogi T Silman AJ Funovits J Felson DT Bingham CO 2010 Rheumatoid arthritis classification criteria: an American College of Rheumatology/European League Against Rheumatism collaborative initiative. *Arthritis Rheum*. (2010) 62:2569–81. 10.1002/art.27584 20872595

[15] Macaubas C Rahman SS Lavi I Haddad A Elias M Sengupta D High dimensional analyses of circulating immune cells in psoriatic arthritis detects elevated phosphorylated STAT3. *Front Immunol*. (2021) 12:758418. 10.3389/fimmu.2021.758418 35087513 PMC8787828

[16] Ando K Hédou JJ Feyaerts D Han X Ganio EA Tsai ES A peripheral immune signature of labor Induction. *Front Immunol*. (2021) 12:725989. 10.3389/fimmu.2021.725989 34566984 PMC8458888

[17] Ellingsen T Hornung N Møller BK Poulsen JH Stengaard-Pedersen K. Differential effect of methotrexate on the increased CCR2 density on circulating CD4 T lymphocytes and monocytes in active chronic rheumatoid arthritis, with a down regulation only on monocytes in responders. *Ann Rheum Dis*. (2007) 66:151–7. 10.1136/ard.2006.054056 16905577 PMC1798497

[18] Eriksson C Rantapää-Dahlqvist S Sundqvist KG. Changes in chemokines and their receptors in blood during treatment with the TNF inhibitor infliximab in patients with rheumatoid arthritis. *Scand J Rheumatol*. (2013) 42:260–5. 10.3109/03009742.2012.754937 23379516

[19] Zhang HH Song K Rabin RL Hill BJ Perfetto SP Roederer M CCR2 identifies a stable population of human effector memory CD4+ T cells equipped for rapid recall response. *J Immunol.* (2010) 185:6646–63. 10.4049/jimmunol.0904156 20980630

[20] Mellado M Martínez-Muñoz L Cascio G Lucas P Pablos JL Rodríguez-Frade JM. T cell migration in rheumatoid arthritis. *Front Immunol*. (2015) 6:384. 10.3389/fimmu.2015.00384 26284069 PMC4515597

[21] Shadidi KR Thompson KM Henriksen JE Natvig JB Aarvak T. Association of antigen specificity and migratory capacity of memory T cells in rheumatoid arthritis. *Scand J Immunol*. (2002) 55:274–83. 10.1046/j.0300-9475.2002.01036.x 11940234

[22] Brühl H Wagner K Kellner H Schattenkirchner M Schlöndorff D Mack M. Surface expression of CC- and CXC-chemokine receptors on leucocyte subsets in inflammatory joint diseases. *Clin Exp Immunol*. (2001) 126:551–9. 10.1046/j.1365-2249.2001.01679.x 11737076 PMC1906244

[23] Künzli M Masopust D. CD4+ T cell memory. *Nat Immunol*. (2023) 24:903–14. 10.1038/s41590-023-01510-4 37156885 PMC10343737

[24] Chang MH Fuhlbrigge RC Nigrovic PA. Joint-specific memory, resident memory T cells and the rolling window of opportunity in arthritis. *Nat Rev Rheumatol*. (2024) 20:258–71. 10.1038/s41584-024-01107-7 38600215 PMC11295581

[25] Chang MH Levescot A Nelson-Maney N Blaustein RB Winden KD Morris A Arthritis flares mediated by tissue-resident memory T cells in the joint. *Cell Rep*. (2021) 37:109902. 10.1016/j.celrep.2021.109902 34706228 PMC8561718

[26] Tormo AJ Letellier MC Sharma M Elson G Crabé S Gauchat JF. IL-6 activates STAT5 in T cells. *Cytokine*. (2012) 60:575–82. 10.1016/j.cyto.2012.07.002 22854263

[27] Lee AW Sharp ER O’Mahony A Rosenberg MG Israelski DM Nolan GP Single-cell, phosphoepitope-specific analysis demonstrates cell type- and pathway-specific dysregulation of Jak/STAT and MAPK signaling associated with in vivo human immunodeficiency virus type 1 infection. *J Virol*. (2008) 82:3702–12. 10.1128/JVI.01582-07 18216116 PMC2268489

[28] Lee J Tam H Adler L Ilstad-Minnihan A Macaubas C Mellins ED. The MHC class II antigen presentation pathway in human monocytes differs by subset and is regulated by cytokines. *PLoS One*. (2017) 12:e0183594. 10.1371/journal.pone.0183594 28832681 PMC5568224

[29] Liang Y Xu WD Peng H Pan HF Ye DQ. SOCS signaling in autoimmune diseases: molecular mechanisms and therapeutic implications. *Eur J Immunol*. (2014) 44:1265–75. 10.1002/eji.201344369 24595859

[30] Cheon H Stark GR. Unphosphorylated STAT1 prolongs the expression of interferon-induced immune regulatory genes. *Proc Natl Acad Sci U S A*. (2009) 106:9373–8. 10.1073/pnas.0903487106 19478064 PMC2688000

[31] Hu X Ivashkiv LB. Cross-regulation of signaling pathways by interferon-gamma: implications for immune responses and autoimmune diseases. *Immunity*. (2009) 31:539–50. 10.1016/j.immuni.2009.09.002 19833085 PMC2774226

[32] Jiao H Li X Li Y Guo Z Yang Y Luo Y Packaged release and targeted delivery of cytokines by migrasomes in circulation. *Cell Discov*. (2024) 10:121. 10.1038/s41421-024-00749-x 39648224 PMC11625823

[33] Luo P Wang P Xu J Hou W Xu P Xu K Immunomodulatory role of T helper cells in rheumatoid arthritis : a comprehensive research review. *Bone Joint Res.* (2022) 11:426–38. 10.1302/2046-3758.117.BJR-2021-0594.R1 35775145 PMC9350707

[34] Aldridge J Ekwall AH Mark L Bergström B Andersson K Gjertsson I T helper cells in synovial fluid of patients with rheumatoid arthritis primarily have a Th1 and a CXCR3+Th2 phenotype. *Arthritis Res Ther*. (2020) 22:245. 10.1186/s13075-020-02349-y 33066816 PMC7566124

[35] Yang WC Hwang YS Chen YY Liu CL Shen CN Hong WH Interleukin-4 supports the suppressive immune responses elicited by regulatory T Cells. *Front Immunol*. (2017) 8:1508. 10.3389/fimmu.2017.01508 29184551 PMC5694475

[36] Jiang Q Yang G Liu Q Wang S Cui D. Function and role of regulatory T cells in rheumatoid arthritis. *Front Immunol*. (2021) 12:626193. 10.3389/fimmu.2021.626193 33868244 PMC8047316

[37] Chu KH Lin SY Chiang BL. STAT6 pathway is critical for the induction and function of regulatory T cells induced by mucosal B Cells. *Front Immunol*. (2020) 11:615868. 10.3389/fimmu.2020.615868 33584704 PMC7878545

[38] Yang Y Day J Souza-Fonseca Guimaraes F Wicks IP Louis C. Natural killer cells in inflammatory autoimmune diseases. *Clin Transl Immunol*. (2021) 10:e1250. 10.1002/cti2.1250 33552511 PMC7850912

[39] Gotthardt D Trifinopoulos J Sexl V Putz EM. JAK/STAT cytokine signaling at the crossroad of NK Cell development and maturation. *Front Immunol*. (2019) 10:2590. 10.3389/fimmu.2019.02590 31781102 PMC6861185

[40] Jackson SH Yu CR Mahdi RM Ebong S Egwuagu CE. Dendritic cell maturation requires STAT1 and is under feedback regulation by suppressors of cytokine signaling. *J Immunol.* (2004) 172:2307–15. 14764699 10.4049/jimmunol.172.4.2307

[41] Karonitsch T von Dalwigk K Steiner CW Blüml S Steiner G Kiener HP Interferon signals and monocytic sensitization of the interferon-γ signaling pathway in the peripheral blood of patients with rheumatoid arthritis. *Arthritis Rheum.* (2012) 64:400–8. 10.1002/art.33347 21953607

[42] Isomäki P Junttila I Vidqvist KL Korpela M Silvennoinen O. The activity of JAK-STAT pathways in rheumatoid arthritis: constitutive activation of STAT3 correlates with interleukin 6 levels. *Rheumatology*. (2015) 54:1103–13. 10.1093/rheumatology/keu430 25406356

[43] Thomas G Tacke R Hedrick CC Hanna RN. Nonclassical patrolling monocyte function in the vasculature. *Arterioscler Thromb Vasc Biol*. (2015) 35:1306–16. 10.1161/ATVBAHA.114.304650 25838429 PMC4441550

[44] Zhang C Li Y Wu Y Wang L Wang X Du J. Interleukin-6/signal transducer and activator of transcription 3 (STAT3) pathway is essential for macrophage infiltration and myoblast proliferation during muscle regeneration. *J Biol Chem*. (2013) 288:1489–99. 10.1074/jbc.M112.419788 23184935 PMC3548462

[45] Kay J Morgacheva O Messing SP Kremer JM Greenberg JD Reed GW Clinical disease activity and acute phase reactant levels are discordant among patients with active rheumatoid arthritis: acute phase reactant levels contribute separately to predicting outcome at one year. *Arthritis Res Ther*. (2014) 16:R40. 10.1186/ar4469 24485007 PMC3978994

[46] Sokka T Pincus T. Erythrocyte Sedimentation Rate, C-Reactive Protein, or rheumatoid factor are normal at presentation in 35%–45% of patients with rheumatoid arthritis seen between 1980 and 2004: analyses from Finland and the United States. *J Rheumatol.* (2009) 36:1387–90. 10.3899/jrheum.080770 19411389

[47] Brown AK Conaghan PG Karim Z Quinn MA Ikeda K Peterfy CG An explanation for the apparent dissociation between clinical remission and continued structural deterioration in rheumatoid arthritis. *Arthritis Rheum*. (2008) 58:2958–67. 10.1002/art.23945 18821687

[48] Gandjbakhch F Conaghan PG Ejbjerg B Haavardsholm EA Foltz V Brown AK Synovitis and osteitis are very frequent in rheumatoid arthritis clinical remission: results from an MRI study of 294 patients in clinical remission or low disease activity state. *J Rheumatol*. (2011) 38:2039–44. 10.3899/jrheum.110421 21885514

[49] Vreju FA Filippucci E Gutierrez M Di Geso L Ciapetti A Ciurea ME Subclinical ultrasound synovitis in a particular joint is associated with ultrasound evidence of bone erosions in that same joint in rheumatoid patients in clinical remission. *Clin Exp Rheumatol.* (2016) 34:673–8. 27192221

[50] Orr CK Najm A Young F McGarry T Biniecka M Fearon U The utility and limitations of CRP, ESR and DAS28-CRP in appraising disease activity in rheumatoid arthritis. *Front Med*. (2018) 5:185. 10.3389/fmed.2018.00185 30123796 PMC6085449

[51] He Z Glass MC Venkatesan P Feser ML Lazaro L Okada LY Progression to rheumatoid arthritis in at-risk individuals is defined by systemic inflammation and by T and B cell dysregulation. *Sci Transl Med*. (2025) 17:eadt7214. 10.1126/scitranslmed.adt7214 40991726 PMC12767604

[52] Tishkowski K Zubair M. *Erythrocyte Sedimentation Rate.* Treasure Island, FL: StatPearls Publishing (2025).

[53] Singh B Goyal A Patel BC. *C-Reactive Protein: Clinical Relevance and Interpretation.* Treasure Island (FL): StatPearls Publishing (2025). 28722873

[54] Tiwari V Jandu JS Bergman MJ. *Rheumatoid Factor.* Treasure Island, FL: StatPearls Publishing (2025).

