# Supplementary material for: Using mass cytometry to probe the STAT signaling landscape in circulating immune cells in Rheumatoid Arthritis uncovers signaling dysregulation and correlation with disease activity
Source: Front Med (Lausanne). 2025 Dec 9;12:1622537. doi: 10.3389/fmed.2025.1622537 (PMC12722927; doi:10.3389/fmed.2025.1622537)
Supplement: Supplementary file 1 [file Data_Sheet_1.docx]

**Using mass cytometry to probe the STAT signaling landscape in circulating immune cells in Rheumatoid Arthritis uncovers signaling dysregulation and correlation with disease activity**

**Claudia Macaubas, Batuhan Bayram, Noor Hussein, Astraea Jager, Kara L Davis, Jonathan Graf, Mary Nakamura, Devy Zisman, Elizabeth D Mellins**


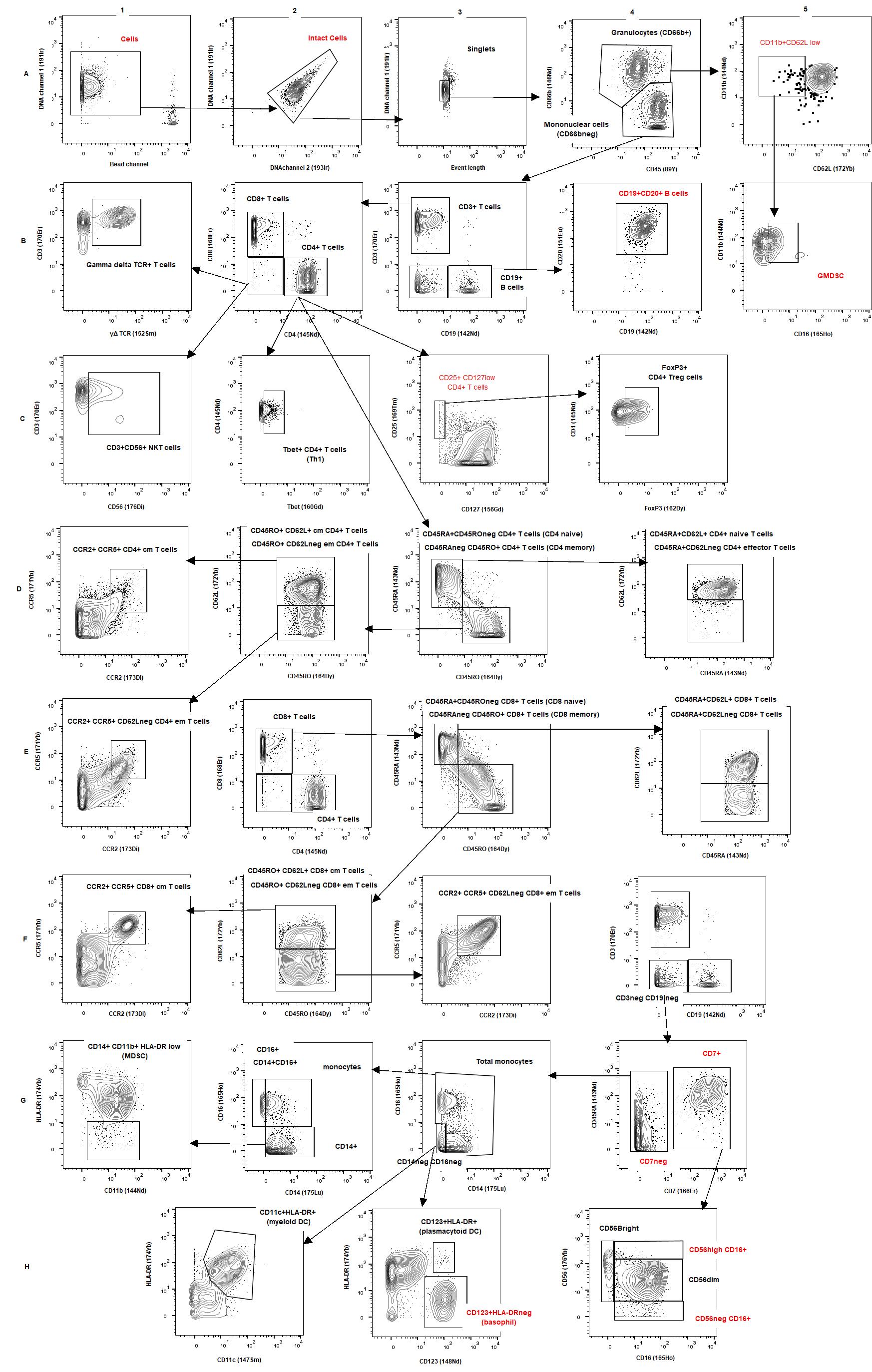


**Supplementary Figure 1.** **Gating strategy to identify immune subpopulations**. The gating strategy was based mostly on Ando et al, 2021. Cell subpopulations labeled in red denote our gating addition or modifications. As we analyzed fixed cells, no live gate was used. The gating strategy is described referring to the column grid (numbered 1 to 5) and the row grid (from A to H). Row A, left to right: **Cells** were gated away from calibration beads using DNA channel 1 and beads; **intact cells** were then selected based on co-staining of DNA 1 and DNA 2, and **singlets** were next selected based on DNA 1 and event length. Next, using CD66b and CD45, the **granulocytes** (CD66b+) and **mononuclear** **cells** (CD66b^neg^) were gated. The CD66b+ population was next analyzed using CD11b and CD62L, and the **CD11b+CD62L^low^** population was further gated using CD11b and CD16 to define granulocytic myeloid-derived suppressor cells (**GMDSC**) (row B, column 5). The CD66b^neg^ population was further gated into the major mononuclear populations using CD3 and CD19: **CD3+ T cells** and **CD19+ B cells** (row B col. 3). The CD19+ B cells was further gated into **CD19+CD20+ B cells** (row B, col. 4). The CD3+ population was gated into **CD4+** and **CD8+** **T cells** (row B, col. 2) and the CD4^neg^CD8^neg^ population was next gated into **TCR gamma delta+ T cells** (row B, col. 1). Using CD3 and CD56, the CD4^neg^CD8^neg^ population was also gated into **CD3+CD56+ NKT cells** (row C, col. 1). The row C also shows the gating of 2 subpopulations of CD4+ T cells: **Tbet+ (Th1 cells)** and **FoxP3+ Treg cells** (CD25+ CD127^low^). On row D, col. 3, the gating of CD4+ T cells memory and naïve subpopulations is shown: **CD4 naïve** (CD45RA+ CD45RO^neg^) and **CD4 memory** (CD45RA^neg^ CD45RO+). The CD4 naïve population was further gated into **CD4+CD45RA+CD62L+** and **CD4+CD45RA+CD62L^neg^** **effector naïve** **T cells** (row D, col. 4). The CD4+ memory population was gated into **CD4+CD45RA+CD62L+** (**CD4+ central memory, cm**) and **CD4+CD45RA+CD62L^neg^** (**CD4+ effector memory, em**) T cells (row D, col. 2). The CD4+CD45RA+CD62L+ cm cells were further gated into **CD4+cmCCR2+CCR5+** cells (Row D, col. 1), and the CD45RA+CD62L^neg^ em cells into **CD4+emCCR2+CCR5+** cells (Row E, col. 1). Row E shows the gating of the **CD8+** T cells (col. 2) into **memory and naïve** subpopulations (col. 3). The CD8 naïve population was further gated into **CD8+CD45RA+CD62L+** and **CD8+CD45RA+CD62L^neg^ effector naïve** T cells (row E, col. 4). The CD8+ memory population was gated into **CD8+CD45RA+CD62L+ (central memory, cm**) and **CD8+CD45RA+CD62L^neg^ (effector memory, em**) memory T cells (row F, col. 2). The CD8+CD45RA+CD62L+ cm cells were further gated into **CD8+cmCCR2+CCR5+ cells** (Row F, col. 1), and the CD8+CD45RA+CD62L^neg^ em cells into **CD8+emCCR2+CCR5+ cells** (Row F, col. 3). Row F shows again the CD3^neg^CD19^neg^ population, which was gated into CD7+ and CD7^neg^ on row G, col. 4. The CD7^neg^ population is gated into **total monocytes** using CD14 and CD16 (row G, col. 3). The total monocytes were next gated into **CD14+, CD14+CD16+ and CD16+** subsets (row G, col. 2). The CD14+ monocytes were further gated into monocytic **MDSC** using HLA-DR and CD11b (row G, col. 1). The CD7+ cells were gated into **NK cells** using CD56 and CD16: **CD56bright, CD56highCD16+, CD56dim, CD56neg CD16+** (row H, col. 3). Row H shows the gating of the CD14^neg^CD16^neg^ population into CD123+HLA-DR+ (**plasmacytoid Dendritic cells**, pDC) and CD123+ HLA-DR^neg^ (**basophils**) (col. 2) and CD11c+HLA-DR+ (**myeloid DC**, col. 1).

**Supplementary Figure 2. Similar distribution of circulating CD4 and CD8 T cells subpopulations between samples from Rheumatoid Arthritis (n=21) and Control subjects (n=10).** Middle line represents the median. Comparisons between all cell subpopulations from RA and control groups were performed using multiple Mann-Whitney tests, and the resulting p-values were corrected using the Benjamini-Hochberg procedure to control the False Discovery Rate [FDR(q)]. All shown comparisons between cell subpopulations from RA patients and controls were deemed non-significant.

**Supplementary Figure 3. Similar distribution of circulating γδ T cells, Natural Killer T (NKT) cells, B cells, and Natural Killer (NK) cells subpopulations between samples from Rheumatoid Arthritis (n=21) and Control subjects (n=10).** Middle line represents the median. Comparisons between all cell subpopulations from RA and control groups were performed using multiple Mann-Whitney tests, and the resulting p-values were corrected using the Benjamini-Hochberg procedure to control the False Discovery Rate [FDR(q)]. All shown comparisons between cell subpopulations from RA patients and controls were deemed non-significant.

**Supplementary Figure 4. Similar distribution of circulating monocytes, myeloid-derived suppressor cells (MDSCs), myeloid dendritic cells (mDCs) and plasmacytoid dendritic cells (pDCs) subpopulations between samples from Rheumatoid Arthritis (n=21) and Control subjects (n=10).** Middle line represents the median. Comparisons between all cell subpopulations from RA and control groups were performed using multiple Mann-Whitney tests, and the resulting p-values were corrected using the Benjamini-Hochberg procedure to control the False Discovery Rate [FDR(q)]. All shown comparisons between cell subpopulations from RA patients and controls were deemed non-significant.

**Supplementary Figure 5. Similar distribution of circulating neutrophils, granulocytic myeloid-derived suppressor cells (G-MDSCs) and basophils subpopulations between samples from Rheumatoid Arthritis (n=21) and Control subjects (n=10).** Middle line represents the median. Comparisons between all cell subpopulations from RA and control groups were performed using multiple Mann-Whitney tests, and the resulting p-values were corrected using the Benjamini-Hochberg procedure to control the False Discovery Rate [FDR(q)]. All shown comparisons between cell subpopulations from RA patients and controls were deemed non-significant.
