# Supplementary material for: Using mass cytometry to probe the STAT signaling landscape in circulating immune cells in Rheumatoid Arthritis uncovers signaling dysregulation and correlation with disease activity
Source: Front Med (Lausanne). 2025 Dec 9;12:1622537. doi: 10.3389/fmed.2025.1622537 (PMC12722927; doi:10.3389/fmed.2025.1622537)
Supplement: Supplementary file 2 [file Table_1.docx]

**Supplementary Table 1.** Signal transducer and activator of transcription (STAT) activators and relevance to immunopathology of Rheumatoid Arthritis (RA)

| **STAT** | **Activated by** | **Relevance to Rheumatoid Arthritis** |
| --- | --- | --- |
| STAT1 | type I and type II interferons (IFNs) (Tolomeo et al, 2022), IL-2, IL-6, IL-7, IL-21, TNF, angiotensin 2, epidermal growth factor (EGF), platelet-derived growth factor (PDGF), hepatocyte growth factor (HGF) (Balendran et al, 2023) | - Induces differentiation of CD4+ T helper cells into Th1 cells, which produce IFNƴ, TNF and IL-2, among others  - These cytokines are involved in synovial inflammation, cartilage destruction and bone erosion (Luo et al, 2022)  - Induces M1-type response (pro-inflammatory) in macrophages; M1-macrophages induced inflammatory response in experimental arthritis, including cartilage destruction  - Increased levels of pSTAT1 in circulating monocytes (Tucci et al, 2022) and/or lymphocytes (Kuuliala et al, 2016) might predict response to JAK inhibitor  - Higher expression of STAT1 in CD8+ T cells in leukocyte rich synovia (Zhang et al, 2019)  - In synovial macrophages, activates CXCL9 and CXCL10 in synovial macrophages, attracting T cells to the synovium (Balendran et al, 2023)  - regulates expression of MMP3 and MMP13, affecting cartilage degradation (Balendran et al, 2023) |
| STAT3 | IL-6 cytokine family (IL-6, IL-11, IL-27, IL-31, CNTF, OSM, and LIF), the IL-10 cytokine family (IL-10, IL 19, IL-20, IL-22, IL-24, IL-26, IL-28A, IL-28B and IL-29), GM-CSF, IL-2, IL-7, IL-21, IFNa/b, and leptin (Balendran et al, 2023) | - Required for CD4+ Th17 differentiation and T follicular helper (Tfh) cells, which help B cells to produce antibodies  - Can inhibit the function of STAT1 (Balendran et al, 2023)  - Participates in Th2 induction  - Regulates MMPs activation in synovial fibroblasts in RA (Araki et al, 2016)  - Promotes osteoclastogenesis |
| STAT4 | IL-12, IL-23, type I interferons (IFNa/b); IL-2 (in NK cells); IL-27, IL-35 (Balendran et al, 2023) (Yang et al, 2020) | - Required for the differentiation of CD4+Th1 cells  - Th1 cells mainly produce IFNƴ, TNF and IL-2, pro-inflammatory cytokines which promote cartilage destruction and bone erosion (Luo)(2)  - Important for Th17 function (secretion of IL-17 in response to IL-23) (Glosson-Byers et al, 2014)  - Genetic polymorphism is associated with increased risk for RA (and SLE) (Remmers et al, 2007) |
| STAT5 | IL-3, the IL-2 cytokine family (IL-2, IL-4, IL-7, IL-9, IL-15 and IL-21), prolactin, EGF, GM-CSF, PDGF and Growth hormone (Balendran et al, 2023) | - Important in the differentiation of Th1, Th2 and Treg; inhibits Th17 and Tfh development (Owen and Farrar, 2017)  - Activation by GM-CSF leads to production of CCL17, a chemokine involved in inflammatory arthritis (Balendran et al, 2023) |
| STAT6 | IL-4 and IL-13 (Balendran et al, 2023) | - Required for the differentiation of CD4+Th2 cells, which secrete IL-4 and IL-13;  - Regulates Treg development and function  - Required for the M2 (anti-inflammatory) activation of macrophages  - In experimental arthritis, Th2-type response has been shown to have anti-inflammatory and anti-osteoclastogenic properties (Luo et al, 2022)  - Th2-type response may be part of an initial regulatory role in RA, and lost once disease is established (Luo et al, 2022)  - pSTAT6 lowering from baseline in lymphocytes associated with good response to treatment (Kuuliala et al, 2016)  - Subsets of Th2 cells (for example, CXCR3+Th2) may play a pathogenic role in synovial inflammation in RA (Aldridge et al, 2020)  - STAT6 overexpressed in synovial tissue and fibroblast-like synoviocytes (FLSs) from RA patients; dowregulation in RA FLSs led to less proliferation, lower inflammatory response and higher apoptosis (Liu et al, 2022) |

**References:**

1. Tolomeo M, Cavalli A, Cascio A. STAT1 and Its Crucial Role in the Control of Viral Infections. Int J Mol Sci. 2022 Apr 7;23(8):4095.

2. Balendran T, Lim K, Hamilton JA, Achuthan AA. Targeting transcription factors for therapeutic benefit in rheumatoid arthritis. Front Immunol. 2023;14:1196931.

3. Yang C, Mai H, Peng J, Zhou B, Hou J, Jiang D. STAT4: an immunoregulator contributing to diverse human diseases. Int J Biol Sci. 2020 Mar 5;16(9):1575–85.

4. Luo P, Wang P, Xu J, Hou W, Xu P, Xu K, et al. Immunomodulatory role of T helper cells in rheumatoid arthritis : a comprehensive research review. Bone Joint Res. 2022 Jul;11(7):426–38.

5. Tucci G, Garufi C, Pacella I, Zagaglioni M, Pinzon Grimaldos A, Ceccarelli F, et al. Baricitinib therapy response in rheumatoid arthritis patients associates to STAT1 phosphorylation in monocytes. Front Immunol. 2022;13:932240.

6. Kuuliala K, Kuuliala A, Koivuniemi R, Kautiainen H, Repo H, Leirisalo-Repo M. STAT6 and STAT1 Pathway Activation in Circulating Lymphocytes and Monocytes as Predictor of Treatment Response in Rheumatoid Arthritis. PLoS One. 2016;11(12):e0167975.

7. Zhang F, Wei K, Slowikowski K, Fonseka CY, Rao DA, Kelly S, et al. Defining inflammatory cell states in rheumatoid arthritis joint synovial tissues by integrating single-cell transcriptomics and mass cytometry. Nat Immunol. 2019 Jul;20(7):928–42.

8. Araki Y, Tsuzuki Wada T, Aizaki Y, Sato K, Yokota K, Fujimoto K, et al. Histone Methylation and STAT-3 Differentially Regulate Interleukin-6–Induced Matrix Metalloproteinase Gene Activation in Rheumatoid Arthritis Synovial Fibroblasts. Arthritis & Rheumatology. 2016;68(5):1111–23.

9. Glosson-Byers NL, Sehra S, Kaplan MH. STAT4 is required for IL-23 responsiveness in Th17 memory cells and NKT cells. JAKSTAT. 2014 Oct 30;3(3):e955393.

10. Remmers EF, Plenge RM, Lee AT, Graham RR, Hom G, Behrens TW, et al. STAT4 and the Risk of Rheumatoid Arthritis and Systemic Lupus Erythematosus. New England Journal of Medicine. 2007 Sep 6;357(10):977–86.

11. Owen DL, Farrar MA. STAT5 and CD4 + T Cell Immunity. F1000Res. 2017 Jan 11;6:32.

12. Aldridge J, Ekwall AKH, Mark L, Bergström B, Andersson K, Gjertsson I, et al. T helper cells in synovial fluid of patients with rheumatoid arthritis primarily have a Th1 and a CXCR3+Th2 phenotype. Arthritis Research & Therapy. 2020 Oct 16;22(1):245.

13. Liu J, Xue J, Xu B, Yu J, Zhang Y, Qin L, et al. miR-135a-5p mediated down-regulation of STAT6 inhibits proliferation and induces apoptosis of fibroblast-like synoviocytes in rheumatoid arthritis. Am J Transl Res. 2022 May 15;14(5):3092–103.
