# Supplementary material for: Using mass cytometry to probe the STAT signaling landscape in circulating immune cells in Rheumatoid Arthritis uncovers signaling dysregulation and correlation with disease activity
Source: Front Med (Lausanne). 2025 Dec 9;12:1622537. doi: 10.3389/fmed.2025.1622537 (PMC12722927; doi:10.3389/fmed.2025.1622537)
Supplement: Supplementary file 4 [file Table_3.docx]

**Supplementary Table 3. Antibody panel for CyTOF**

| **Target** | **Label** | **Target**  **location** | **Clone** | **Source – Catalog number** | **Amount used (μl)/sample** | **RRID^1^** |
| --- | --- | --- | --- | --- | --- | --- |
| CD45 | 89Y | Surface | HI30 | SB^2^ – 3089003B | 1 | AB_2938863 |
| CD354 (Trem1)^3^ | 141Pr | Surface | Trem-26 | BioLegend - 314902 | 1 | AB_389213 |
| CD19 | 142Nd | Surface | HIB19 | SB – 3142001B | 1 | AB_3661857 |
| CD45RA | 143Nd | Surface | HI100 | SB – 3143006B | 0.8 | AB_2651156 |
| CD11b (Mac-1) | 144Nd | Surface | ICRF44 | SB – 3144001B | 1 |  |
| CD4 | 145Nd | Surface | RPA-T4 | SB – 3145001B | 1 | AB_3661845 |
| CD66b^4^ | 146Nd | Surface | G10F5 | BioLegend -305102 | 0.7 | AB_314494 |
| CD11c | 147Sm | Surface | Bu15 | SB – 3147008B | 0.9 | AB_3665423 |
| CD123 (IL-3R)^4^ | 148Nd | Surface | 6H6 | BioLegend - 306027 | 0.9 | AB_2562823 |
| pStat4 [Y693]^4^ | 149Sm | Intracellular (phospho) | Rabbit IgG polyclonal | Invitrogen - 71-7900 | 1 | AB_2533998 |
| pStat5 [Y694] | 150Nd | Intracellular (phospho) | 47 | SB – 3150005A | 1 | AB_2744690 |
| CD20^4^ | 151Eu | Surface | 2H7 | BioLegend - 302343 | 1 | AB_2562816 |
| TCRgd | 152Sm | Surface | 11F2 | SB – 3152008B | 1 | AB_2687643 |
| pStat1 [Y701] | 153Eu | Intracellular (phospho) | 4a | SB – 3153005A | 1 | AB_2744689 |
| CD33^4^ | 154Sm | Surface | P67.6 | BioLegend – 825601 | 0.9 | AB_2564890 |
| CD115^5^  (CSF-1R) | 155Gd | Surface | SKII.4 | BioLegend - 337602 | 1 | AB_2300508 |
| CD127 (IL-7Ra)^4^ | 156Gd | Surface | R34-34 | Novus Bio - DDX0700P-100 | 0.9 |  |
| pStat3 [Tyr705] | 158Gd | Intracellular (phospho) | 4/P-Stat3 | SB – 3158005A | 1 | AB_2811100 |
| Tbet | 160Gd | Intracellular | 4B10 | SB – 3160010B | 1 | AB_2810251 |
| pStat6 [Y641]^4^ | 161Dy | Intracellular (phospho) | A15137E | BioLegend 686002 | 1 | AB_2616820 |
| FoxP3 | 162Dy | Intracellular | PCH101 | SB – 3162011A | 1 |  |
| CD69^4^ | 163Dy | Surface | FN50 | BioLegend - 310939 | 1 | AB_2562827 |
| CD45RO | 164Dy | Surface | UCHL1 | SB – 3164007B | 0.9 | AB_2811092 |
| CD16 | 165Ho | Surface | B73.1 | SB – 3165007B | 1 |  |
| CD7 | 166Er | Surface | M-T701 | SB – 3166027B | 0.75 |  |
| CD8a | 168Er | Surface | SK1 | SB – 3168002B | 1 | AB_2892771 |
| CD25 (IL-2R) | 169Tm | Surface | 2A3 | SB – 3169003B | 1 | AB_2938861 |
| CD3 | 170Er | Surface | UCHT1 | SB – 3170001B | 0.9 | AB_2811085 |
| CD195 (CCR5) | 171Yb | Surface | NP-6G4 | SB – 3171017A | 1 | AB_3676415 |
| CD62L (L-selectin)^4^ | 172Yb | Surface | DREG.200 | Thermo Scientific BMS1015 | 0.75 | AB_10596353 |
| CD192 (CCR2)^4^ | 173Yb | Surface | K036C2 | Antibody: Biolegend – 357202 | 1 | AB_2561851 |
| HLA-DR | 174Yb | Surface | L243 | SB – 3174001B | 1 |  |
| CD14 | 175Lu | Surface | M5E2 | SB – 3175015B | 1 | AB_2811083 |
| CD56 (NCAM) | 176Yb | Surface | NCAM16.2 | SB – 3176008B | 0.9 | AB_2938870 |

^1^: RRID: Research Resource Identifiers; ^2^: SB: Standard BioTools; ^3^: in house labeling with SB MaxPar kit X8 – 201141A; ^4^: in house labeling with SB MaxPar kit X8 Multimetal – 201300; ^5^: in house labeling with SB MaxPar kit X8 201155B
